# *PPARG2* Pro12Ala polymorphism influences body composition changes in severely obese patients consuming extra virgin olive oil: a randomized clinical trial

**DOI:** 10.1186/s12986-018-0289-4

**Published:** 2018-07-17

**Authors:** Ana Paula Santos Rodrigues, Lorena Pereira Souza Rosa, Erika Aparecida Silveira

**Affiliations:** Programa de Pós-Graduação em Ciências da Saúde, Faculdade de Medicina, Universidade Federal de Goiás, 1a Avenida, s/n, Setor Leste Universitário, Goiânia, Goiás CEP 74605-020 Brazil

**Keywords:** PPAR gamma, Interleukin-6, Weight loss, Diet, Body composition, Adiposity

## Abstract

**Background:**

Previous intervention studies have reported the association of the *PPARG2* Pro12Ala (rs1801282) and *IL6* -174G > C (rs1800795) polymorphisms with weight loss; however, their results are inconsistent. We aimed to investigate the effect of the *PPARG2* Pro12Ala and *IL6* -174G > C polymorphisms on body weight, body composition and metabolic parameters after a 12-week nutritional intervention with a traditional Brazilian diet and extra virgin olive oil supplementation in severely obese patients.

**Methods:**

A total of 149 severely obese individuals [body mass index (BMI) ≥ 35 kg/m^2^] were randomized into three 12-week nutritional intervention groups – the extra virgin olive oil supplementation (OO) group (*n* = 50), the traditional Brazilian diet (DieTBra) group (*n* = 49), and the DieTBra plus extra virgin olive oil supplementation (DieTBra+OO) group (n = 50). Anthropometric measurements, body composition, metabolic parameters, physical activity practise and dietary intake were assessed. The associations were tested using generalized linear models adjusted for confounders.

**Results:**

The *PPARG2* Pro12Ala polymorphism influenced body composition changes. Ala carriers in the intervention groups with extra virgin olive oil supplementation had greater reductions in the percentage of body fat (%BF) (OO: *p* = 0.049, DietBra+OO: *p* = 0.004) and greater increases in both fat-free mass (FFM) (OO: *p* = 0.020, DieTBra: *p* = 0.007) and lean mass (LM) (OO: p = 0.020, DieTBra+OO: p = 0.007) than did ProPro homozygotes. No association was found for the *IL6* -174G > C polymorphism.

**Conclusions:**

Extra virgin olive oil intake may modulate favourable body composition changes, promoting a decrease in the %BF and increases in the LM and FFM of severely obese individuals, even without weight loss, in the presence of the Ala allele of the Pro12Ala polymorphism.

**Trial registration:**

Registered under ClinicalTrials.gov Identifier No. NCT02463435.

**Electronic supplementary material:**

The online version of this article (10.1186/s12986-018-0289-4) contains supplementary material, which is available to authorized users.

## Background

An obesogenic environment has an overriding importance in the development of obesity; however, genetic susceptibility also plays a critical role [1]. Different responses to weight loss interventions among individuals may be related to genetic variations [2, 3]. In this sense, many polymorphisms of genes involved in adipogenesis, lipid metabolism, energy expenditure, and the regulation of appetite and food intake have been studied [4, 5]. The Pro12Ala (rs1801282) polymorphism, which is the most studied polymorphism in the peroxisome proliferator-activated receptor γ gene (*PPARG2*), and the -174G > C (rs1800795) polymorphism in the promoter region of the interleukin-6 gene (*IL6*) have been reported to influence weight loss treatment outcomes [2, 6].

The *PPARG2* gene controls the expression of genes involved in adipocyte differentiation, fatty acid glucose metabolism, and inflammatory processes [7]. The Ala allele of the Pro12Ala polymorphism has been associated with elevated BMI, especially among severely obese individuals [8]. Increasing amounts of data on the Pro12Ala polymorphism have shown that gene-diet interactions are responsible for the weight loss variability in intervention studies, partly in conjunction with fatty acid intake [9].

The -174G > C polymorphism (rs1800795) has been shown to influence the transcriptional regulation and plasma levels of the pro-inflammatory cytokine IL-6 [10, 11]. However, the association between the C allele and increased BMI is inconsistent [12, 13]. Intervention studies have reported the association of the C allele with greater weight reduction in individuals with high cardiovascular risk [14], protection against weight regain in obese individuals, and improvement in the ability to maintain weight in the presence of the Pro12Ala Ala allele [15]. Nevertheless, the C allele also induced resistance to weight loss in morbidly obese individuals after laparoscopic gastric banding [16].

Recently, weight loss intervention studies have focused on healthy eating patterns, such as the Mediterranean Diet (MedDiet). In addition to cardiometabolic benefits, MedDiet has also been associated with weight loss and lower BMI, and olive oil is one of the most important components of this eating pattern [17]. In Brazil, the traditional eating pattern, which is based on the consumption of rice and beans, is also considered healthy and is associated with lower levels of overweight and obesity [18–20]. The consumption of olive oil has been increasing not only in Brazil but also in other countries [21], and the effects of olive oil on other dietary patterns should be investigated.

Given the controversial results about the impact of the Pro12Ala and -174G > C variants on the weight loss response to lifestyle interventions and the need for more effective nutritional strategies to fight severe obesity, randomized intervention trials may provide the strongest evidence in this matter. Thus, the aim of the current study was to assess the effect of the *PPARG2* Pro12Ala and *IL6* -174G > C polymorphisms on the body weight, body composition and metabolic parameters of severely obese patients after a 12-week nutritional intervention with a traditional Brazilian diet and extra virgin olive oil supplementation. Moreover, the association of the aforementioned polymorphisms with diet-induced weight loss has been poorly investigated in severely obese individuals.

## Methods

### Study design and subjects

Severely obese patients (BMI ≥ 35 kg/m^2^) were investigated according to a parallel group, single-blinded, controlled randomized clinical trial with nutritional intervention and extra virgin olive oil supplementation (DieTBra Trial) for 12 weeks. The study was conducted from June 2015 to February 2016 in Goiânia, Goiás State, Brazil, at the Nutrition in Severe Obesity Outpatient Clinic of the Clinical Research Unit of the Clinical Hospital/Federal University of Goiás.

All participants provided written informed consent prior to enrolment. The study protocols were performed in accordance with the ethical standards specified in the 1961 Declaration of Helsinki (as revised in Hong Kong in 1989, in Edinburgh in 2000, and in South Korea in 2008) and were approved by the Ethics Committee on Research with Humans of the Clinical Hospital/Federal University of Goiás under protocol number 747.792. The trial was also registered at ClinicalTrials.gov (NCT02463435).

Our study included patients with a BMI ≥ 35 kg/m^2^ and an age between 18 and 65 years who were referred to our outpatient clinic from the primary care of the Brazilian Unified Health System (SUS) in Goiânia. SUS is a public health care system designed to offer free and universal health coverage in Brazil and is divided into basic, specialized and high complexity categories of assistance. We excluded volunteers who had already undergone bariatric surgery; had been under nutritional treatment for weight loss within the previous 2 years; were currently taking anti-obesity or anti-inflammatory drugs; had experienced a weight loss higher than 8% in the last 3 months; had been diagnosed with HIV/AIDS or had heart/kidney/hepatic insufficiency, chronic obstructive pulmonary disease, or cancer; or were currently pregnant.

### Baseline, randomization, and blinding

After enrolment, patients underwent a baseline phase that was divided into two parts due to the large amount of data to be collected and to allow the assessment of physical activity by an accelerometer for seven consecutive days. In baseline part 1, we collected sociodemographic, clinical, and anthropometrical data. At that time, the accelerometer was also fastened onto the patients’ wrist. In baseline part 2, body composition and dietary intake were assessed, blood samples were drawn from each participant, and the accelerometer was retrieved. At the end of the baseline phase, the study participants were randomly assigned by the same trained researcher into three intervention groups in 1:1:1 ratio using an algorithm available at www.randomization.com.

**Fig. 1:**
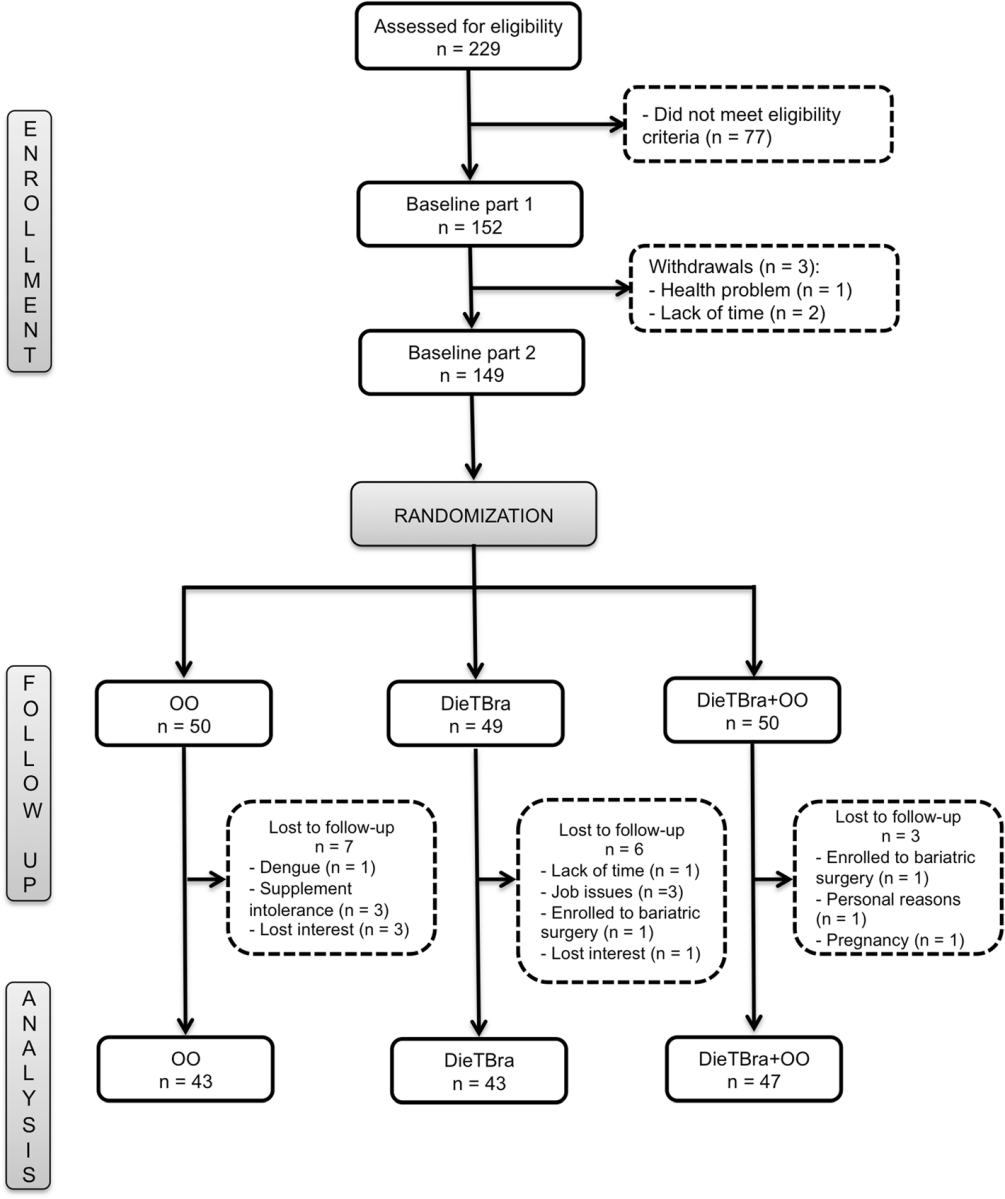
Flow chart of the study participants

Randomization resulted in the placement of the participants into three groups, namely: 1) the olive oil supplementation (OO) group, which received extra virgin olive oil supplementation of 52 mL/d, 2) the traditional Brazilian diet (DieTBra) group, which received nutritional intervention for weight loss, and 3) the traditional Brazilian diet plus olive oil supplementation (DieTBra+OO) group, which received the same intervention as the DieTBra group in addition to extra virgin olive oil supplementation of 52 mL/d. Despite the difficulties of and barriers to blinding dietary interventions in clinical trials [22], in this study, patients were blinded to the type of supplement consumed. To assure blinding, patients in the OO and DieTBra+OO groups were told that the sachets of extra virgin olive oil were a dietary supplement enriched with bioactive compounds in an oily form. The information on the supplement sachets was adequately prepared according to the recommendations of the National Health Surveillance Agency of Brazil for Clinical Trials to mask this intervention. The research team was trained to use the term “dietary supplement enriched with bioactive compounds” or just “nutritional supplement” to describe the olive oil to the patients in the groups that received the supplement. Furthermore, the follow-up visits of each intervention group occurred on a different day of the week to avoid contact among the participants. Fig. 1 shows the flow chart of the participants in the study.

### Interventions and follow-up visits

The DieTBra nutritional intervention comprises a restricted energy food plan for weight loss. The formulation of the food plan was based on the traditional Brazilian diet, which consisted of rescuing the healthy Brazilian eating habits used by the general population in Brazil before the nutritional transition occurred. The nutritional transition changed the food pattern by including fast food and large amount of ultra-processed foods in the diet. DieTBra includes rice and beans, along with a small portion of meat or poultry, and fresh raw and cooked vegetables in the main meals. Fruits are common at breakfast and at other smaller meals; dairy products, bread and eggs are also common [23, 24]. Furthermore, the recommendations of the new Food Guide for the Brazilian Population were applied. All patients were encouraged to eat fresh and/or minimally processed foods instead of ultra-processed foods [24]. An individualized and balanced food plan was prescribed; the plan was divided in four to six meals a day and considered cultural, social, and economic reality as well as aspects of eating behaviour [24].

We used a specific equation for severely obese patients to calculate resting energy expenditure [25]. Total energy expenditure was determined using physical activity level factors [26], and the thermic effect of food [27]. Total food plan energy was determined based on a weight reduction goal for the 12-week follow-up according to the patient’s BMI (5 to 10% weight loss), resulting in a daily restriction of 550 to 1100 kcal. Macronutrient distribution followed the Dietary Reference Intake (DRI) recommendations: 45–65% carbohydrates, 10–35% proteins, and 20–35% lipids [26].

Additionally, groups prescribed DieTBra also received nutritional instructions according to metabolic disturbances and comorbidities and instructions about physical activity practice according to the World Health Organization’s recommendation (at least 150 min of moderate-intensity aerobic physical activity per week) [28].

Patients in the OO group received only the extra virgin olive oil supplementation (52 mL/d divided into 4 sachets of 13 mL each) and were instructed to maintain their usual diet. The DieTBra+OO group received the nutritional intervention as above plus extra virgin olive oil supplementation (52 mL/d in 4 sachets of 13 mL each). The total food plan energy for the DieTBra+OO patients was adjusted to include the extra virgin olive oil supplement.

At the end of each visit, patients were provided, at no cost, a sufficient quantity of extra virgin olive oil sachets to last until the next visit (4 weeks). Patients were instructed about the consumption of the extra virgin olive oil (preferably at lunch and dinner, not to be used for cooking, and not to be shared with others). To control the consumption of the extra virgin olive oil, patients were asked to return all sachets, even the ones not consumed. The intervention lasted 12 weeks, and visits took place every 4 weeks. Trained registered dietitians delivered the interventions. Standard operating procedures were developed to train the entire research team on the measurements and assessments performed throughout the study, and periodic training and group meetings were conducted to assure the quality of the data collected.

### Body composition measurements

The anthropometric measurements included height and body weight. Measurements were performed using standardized procedures [29]. BMI was calculated by dividing the body mass (kg) by the square of the height (m^2^). Severe obesity was defined as a BMI ≥ 35 kg/m^2^ [30].

Fat mass (kg), fat-free mass (kg), percentage of body fat (%) and lean mass (kg) were assessed by dual-energy X-ray absorptiometry (DXA) using a Lunar DPX NT device (GE Healthcare, Madison, WI, USA). The measurement criteria were as follows: a 12-h fast prior to measurement, the avoidance of strenuous physical activity and alcohol, as well as food and drinks containing caffeine, on the day prior to measurement, and the absence of pacemakers and orthopaedic prostheses/implants (devices containing metal). DXA assessment was performed on patients lying in a supine position on the scanner table with arms close to the body. Duplication of right arm data using the hemiscan technique was performed when the subject was too large for the equipment to capture both arms [31]. DXA assessment was limited to patients with a body weight up to 130 kg due to the device capacity. Body weight and DXA measurements were performed at baseline part 2 and at week 12.

### Dietary intake

Twenty-four-hour records were used to assess dietary intake in the baseline phase and at final follow-up. Trained registered dietitians collected three 24-h records within 7 days using the Multiple-Pass Method; two were collected face-to-face and one was collected over the phone [32]. A nutritional analysis was performed using Avanutri Online (Avanutri Equipamentos de Avaliação Ltda, Rio de Janeiro, RJ, BR). Energy (kcal), proteins (%), carbohydrates (%), lipids (%), saturated fatty acids (SAF) (%), monounsaturated fatty acids (MUFA) (%), polyunsaturated fatty acids (PUFA) (%), and the polyunsaturated:saturated fatty acids ratio (P:S ratio) were obtained by calculating the means of these parameters from the three 24-h records.

### Physical activity assessment

We measured physical activity objectively using the tri-axial accelerometer ActiGraph wGT3X (ActiGraph, Pensacola, FL, USA). At baseline and at final follow-up, study participants were instructed to wear the accelerometer on the non-dominant wrist 24 h a day, even when showering and at bedtime, for seven consecutive days. The device was programmed by the researcher to capture data from midnight starting the day after the allotment until midnight ending the day before the collection, thus capturing six full days of data. The wGT3X measured the acceleration in three axes (x, y, z) within a dynamic range of ±8 g and a sampling frequency of 30 Hz.

We used ActiLife 6.11.7 software to initialize and download the accelerometer data. Individuals were included in the analysis if data were available for at least 50% of the wear time. Output data were processed using the R package GGIR (http://cran.r-project.org). Vector magnitude of activity-related acceleration (three axes) was calculated using the Euclidian norm minus 1 g (ENMO: √x^2^ + y^2^ + z^2^–1 g). Activity intensity was estimated as the average time per day spent in moderate and vigorous physical activity from 5-s aggregated time series. The outcomes used in the present study were moderate-to-vigorous physical activity (MVPA), which was considered to include activities with an acceleration equal to or greater than 100 mg [33] with an estimated time spent equal to or greater than 10 min per bout during a week; and sedentary time, which was considered to include activities with an acceleration lower than 50 mg (non-bouted) per day. Bouts of MVPA were defined as 10-min time windows that started with a 5-s epoch value equal to or greater than 100 mg and for which 80% of the subsequent 5-s epoch values were equal to or greater than the 100 mg threshold [34].

### Biochemical analysis

Blood samples for biochemical analysis and genotyping were collected after 12-h overnight fasting at baseline and at week 12. Serum glucose, total cholesterol, high-density lipoprotein (HDL), low-density lipoprotein (LDL), and triglycerides were measured by enzyme-colorimetric methods. Serum insulin was measured by chemiluminescence, and haemoglobin A1c (HbA1c) was measured by liquid chromatography. The homeostasis model assessment of insulin resistance (HOMA-IR) was calculated following the formula of Matthews et al. [35].

### DNA extraction and genotyping

DNA was extracted from whole blood using a commercial kit (PureLink™ Genomic DNA Mini Kit; Invitrogen, Carlsbad, CA, USA). DNA concentration and purity were evaluated by spectrophotometric determination of the A_260/280_ ratio using a NanoDrop® 2000c (Thermo Fisher Scientific, Waltham, MA, USA). DNA quality was assessed using agarose gel electrophoresis. Genotyping of *PPARG2* (Pro12Ala, rs1801282) and *IL6* (−174G > C, rs1800795) was carried out using a TaqMan® SNP Genotyping assay (Applied Biosystems, Foster City, CA, USA) for each polymorphism with a StepOne Real-Time PCR system (Thermo Fisher Scientific, Waltham, MA, USA). For quantitative polymerase chain reaction (qPCR), a TaqMan GTExpress™ Master Mix (Thermo Fisher Scientific, Waltham, MA, USA) reagent kit was used, and qPCR was performed in a final volume of 21 μL according to the manufacturer’s instructions. Although DNA samples were extracted for all study participants, the qPCR amplification was only conducted for the *PPARG2* Pro12Ala polymorphism on samples from 145 individuals and for the *IL6* -174G > C polymorphism on samples from 147 individuals.

### Statistical analysis

We structured the dataset in EpiData 3.1 with double entry typing and validation for the assessment of consistency. Data are expressed as the means ± SD for continuous variables and as frequencies and percentages for categorical variables. The Kolmogorov-Smirnov test was used to analyse the distribution of continuous data. The homogeneity among the nutritional intervention groups was tested using analysis of variance (ANOVA) or a Kruskal-Wallis test and a Chi-squared test. The Hardy-Weinberg equilibrium was tested in the population using a Chi-squared test. The mean daily extra virgin olive oil intake was compared between the OO and DieTBra+OO groups using Student’s t-test. The delta (12th week measurement – baseline measurement) was calculated for food intake variables. Comparison of food intake means was performed using a Wilcoxon signed-rank test and a Kruskal-Wallis test followed by post hoc Bonferroni correction.

We investigated the changes in the anthropometric and body composition measurements as primary outcomes and the changes in the metabolic markers as secondary outcomes. General linear models (GLMs) were performed to test the main effects of the polymorphisms and nutritional interventions on the outcomes. Models were adjusted for potential confounders such as age, sex, baseline BMI and sedentary time. Multiplicative interaction terms (polymorphism and sex-by-nutritional intervention) were used in the models to test the statistical homogeneity of the effects, and as there was no interaction, we examined the main effects after removing the interaction term from the model. When effects were found for a polymorphism, we compared the outcome by genotypes (dominant model) for each nutritional intervention using Student’s t-test.

Data analyses were performed using STATA version 12.0 (StataCorp, College Station, TX, USA), and statistical significance was defined at *P* < 0.05.

## Results

Baseline characteristics of the studied participants are presented in Table 1. As expected, due to randomization, there were no significant differences between the nutritional intervention groups at baseline. The genotype distribution of both polymorphisms did not deviate from the Hardy-Weinberg equilibrium expectation (*p* = 0.689 for *PPARG2* Pro12Ala, and *p* = 0.863 for *IL6* -174G > C). All analyses were conducted with a dominant genetic model because of the small number of AlaAla homozygotes for the Pro12Ala polymorphism and CC homozygotes for the -174G > C polymorphism. The retention rate in the current study was 89%. Olive oil consumption was similar (*p* = 0.731) in OO and DieTBra+OO (40.6 ± 11.1 and 39.8 ± 10.7, respectively).

**Table 1:** Sociodemographic, anthropometric, biochemical and polymorphism characteristics of the study groups

Variable	Total *n* = 149	OO n = 50	DieTBra *n* = 49	DieTBra + OO *n* = 50
Age	39.63 ± 8.82	38.14 ± 8.14	39.14 ± 8.16	41.60 ± 9.85
Sex				
Female	127 (85.2)	45 (90.0)	40 (81.6)	42 (84.0)
Male	22 (14.8)	5 (10.0)	9 (18.4)	8 (16.0)
Years of schooling	9.60 ± 3.05	9.66 ± 2.95	9.86 ± 2.96	9.28 ± 3.26
Social status				
A/B	34 (22.8)	13 (26.0)	10 (20.4)	11 (22.0)
C	64 (43.0)	20 (40.0)	24 (49.0)	20 (40.0)
D/E	51 (34.2)	17 (34.0)	15 (30.6)	19 (38.0)
Weight (kg)	118.81 ± 19.47	117.38 ± 18.69	120.44 ± 20.85	118.64 ± 19.10
Height (m)	1.60 ± 0.07	1.60 ± 0.07	1.61 ± 0.08	1.60 ± 0.06
BMI (kg/m^2^)	46.04 ± 6.41	45.77 ± 6.27	46.22 ± 6.26	46.13 ± 6.79
Fat mass (kg)*	54.05 ± 11.04	52.39 ± 11.77	55.21 ± 8.32	54.37 ± 11.03
Fat free mass (kg)*	53.42 ± 8.04	53.49 ± 7.92	54.16 ± 7.42	52.64 ± 8.81
Body fat percentage (%)*	51.62 ± 5.18	50.56 ± 5.11	51.54 ± 4.70	52.63 ± 5.60
Lean mass (kg)*	51.07 ± 7.93	51.07 ± 7.81	51.84 ± 7.5	50.33 ± 8.60
Fasting glucose (mg/dL)	109.95 ± 45.24	104.44 ± 29.03	107.43 ± 35.15	117.94 ± 63.45
Fasting insulin (μU/mL)	23.42 ± 14.86	25.24 ± 17.74	24.40 ± 14.64	20.65 ± 11.39
HOMA-IR	6.40 ± 4.89	6.78 ± 5.87	6.68 ± 4.76	5.75 ± 3.87
HbA1c (%)	6.30 ± 1.43	6.13 ± 1.27	6.26 ± 1.42	6.52 ± 1.58
Total cholesterol (mg/dL)	189.12 ± 38.10	187.74 ± 37.59	184.31 ± 33.40	195.22 ± 42.61
HDL cholesterol (mg/dL)	47.62 ± 11.35	47.18 ± 10.32	48.45 ± 10.51	47.26 ± 13.16
LDL cholesterol, (mg/dL)	109.44 ± 35.48	106.94 ± 29.96	106.40 ± 32.56	114.76 ± 42.51
Triglyceride (mg/dL)	160.31 ± 78.40	160.46 ± 79.86	154.76 ± 87.76	165.6 ± 67.72
MVPA (min/week)^†^	44.29 ± 61.11	46.85 ± 56.89	49.10 ± 69.57	37.04 ± 56.83
Sedentary time (min/d)	1176.85 ± 83.28	1171.35 ± 73.25	1171.24 ± 81.74	1187.85 ± 8.12
Pro12Ala polymorphism^‡^				
ProPro	126 (86.9)	43 (43.4)	41 (40.8)	42 (41.7)
ProAla+AlaAla	19 (13.1)	7 (6.6)	6 (6.2)	6 (6.3)
Pro alelle	270 (93.1)	92 (92.0)	88 (93.6)	90 (93.8)
Ala alelle	20 (6.9)	8 (8.0)	6 (6.4)	6 (6.2)
-174G > C polymorphism^§^				
GG	96 (65.3)	38 (39.6)	29 (30.2)	29 (30.2)
CG + CC	51 (34.7)	12 (23.5)	18 (35.3)	21 (41.2)
G alelle	238 (81.0)	87 (87.0)	75 (79.8)	76 (76.0)
C alelle	56 (19.0)	13 (13.0)	19 (20.2)	24 (24.0)

There was no difference among the groups for any variable. Data are shown as absolute means ± SD or N (%). OO: olive oil group, DieTBra: traditional Brazilian diet group, DieTBra+OO: traditional Brazilian diet plus olive oil group, BMI: Body Mass Index, HOMA-IR: homeostatic model assessment for insulin resistance, *HbA1c* haemoglobin A1c, *HDL* high-density lipoprotein, *LDL* low-density lipoprotein, *MVPA* moderate-to-vigorous physical activity

*n = 111, OO: *n* = 34, DieTBra: *n* = 38, DieTBra+OO: *n* = 39, ^†^*n* =140, OO: *n* = 47, BraDiet: *n* = 46, BraDiet+OO: *n* = 47, ^‡^*n* = 145, ^§^*n* = 147

Interventions promoted significant changes in the food intake of the study participants. At the end of follow-up, the DieTBra and DieTBra+OO groups had a decreased total energy intake compared to that at baseline. The delta for the total energy intake showed a greater reduction in both of those groups than in the OO group. Considering total fat intake, the DieTBra group had a reduced consumption, while an increased consumption was observed in the OO and DieTBra+OO groups between baseline and final follow-up. The delta for total fat intake was greater in the OO and DieTBra+OO groups than in the DieTBra group. Regarding carbohydrates, PUFA and SAF, we observed significant reductions in the OO and DieTBra+OO groups between baseline and final follow-up, but the delta for these variables was not different between the intervention groups (Table 2).

**Table 2:** Change in energy and macronutrient intake according to the intervention group after 12 weeks of nutritional intervention

	OO			DieTBra			DieTBra+OO		
	Baseline N = 50	Final follow-up *N* = 43	P*	Baseline N = 49	Final follow-up *N* = 43	P*	Baseline *N* = 50	Final follow-up *N* = 47	P*
Total energy intake (kcal/day)	1594.3 ± 580.5	1708.7 ± 545.8	0.147	1661.2 ± 603.8	1219.9 ± 387.4	< 0.001	1814.0 ± 904.7	1395.6 ± 403.6	< 0.001
∆	114.39 ± 561.06^a,b^			− 441.2 ± 573.8^a^			− 418.4 ± 778.2^b^		
Carbohydrates (% total energy)	50.2 ± 9.5	46.7 ± 8.0*	0.044	52.6 ± 9.4	51.7 ± 8.9	0.708	51.3 ± 7.7	45.4 ± 6.7*	< 0.001
∆	− 3.52 ± 11.36			− 0.9 ± 11.0			−5.9 ± 10.4		
Protein (% total energy)	16.9 ± 4.3	15.7 ± 4.1	0.143	17.2 ± 4.8	18.3 ± 4.9	0.209	18.3 ± 5.1	16.8 ± 5.8	0.200
∆	− 1.16 ± 4.56			1.1 ± 6.4			− 1.5 ± 6.8		
Fat (% total energy)	26.7 ± 6.1	34.5 ± 7.6	< 0.001	28.7 ± 7.2	25.3 ± 6.3	0.009	28.3 ± 6.7	35.0 ± 8.5*	< 0.001
∆	7.79 ± 9.74^a^			−3.4 ± 7.6^a,b^			6.7 ± 11.6^b^		
MUFA (% total fat)	7.96 ± 2.98	15.00 ± 7.01	< 0.001	7.75 ± 2.84	6.61 ± 3.13	0.072	7.60 ± 1.97	16.30 ± 6.75	< 0.001
∆	7.04 ± 7.53^a^			− 1.14 ± 3.52^a,b^			8.70 ± 6.98^b^		
PUFA (% total fat)	15.52 ± 5.34	12.92 ± 3.21	0.016	14.54 ± 4.91	14.11 ± 5.68	0.562	15.13 ± 4.46	12.55 ± 3.62	0.009
∆	− 2.60 ± 6.39			− 0.43 ± 6.84			− 2.58 ± 6.20		
SAF (% total fat)	28.59 ± 8.07	24.27 ± 5.07	0.002	31.63 ± 7.75	29.67 ± 7.96	0.346	28.23 ± 6.01	23.44 ± 5.85	< 0.001
∆	− 4.22 ± 8.80			− 1.96 ± 9.84			− 4.80 ± 7.28		
P:S ratio	0.62 ± 0.35	0.55 ± 0.18	0.340	0.51 ± 0.26	0.50 ± 0.22	0.971	0.57 ± 0.23	0.57 ± 0.21	0.949
∆	−0.07 ± 0.38			− 0.01 ± 0.31			0.001 ± 0.31		

Data are shown as absolute means ± SD or N (%). *OO* olive oil group, *DieTBra* traditional Brazilian diet group, *DieTBra + OO* traditional Brazilian diet plus olive oil group, *MUFA* monounsaturated fatty acids, *PUFA* polyunsaturated fatty acids, *SAF* saturated fatty acids, *P:S ratio* PUFA:SAF ratio, ∆: 12th week measurement – baseline measurement

*Wilcoxon signed rank test

Equal letters mean significant difference between groups (*p* < 0.05) compared by Kruskal-Wallis test followed by Bonferroni correction post-hoc test

**Fig. 2:**
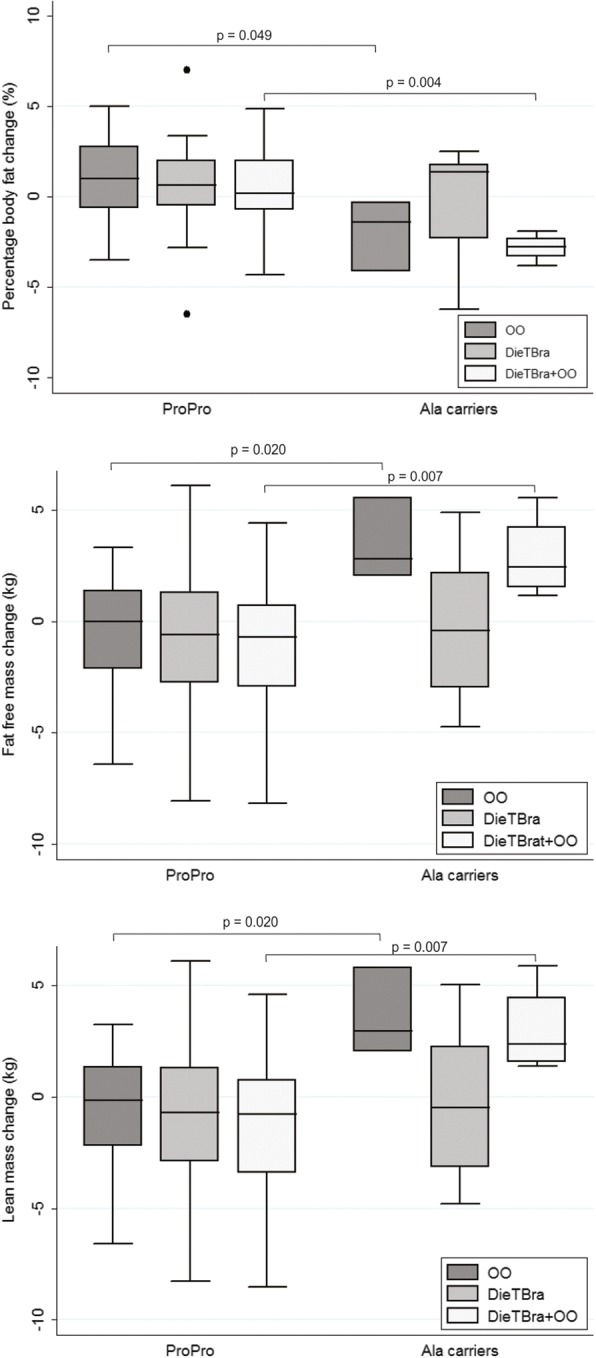
Effect of the *PPARG2* Pro12Ala polymorphism on the body fat percentage, fat-free mass and lean mass of severely obese patients after a 12-week nutritional intervention. DieTBra: traditional Brazilian diet, DieTBra+OO: traditional Brazilian diet plus olive oil supplementation, OO: olive oil supplementation. Dots represent outlier values

Analyses of the effects of the polymorphisms and nutritional interventions on anthropometric measurements, body composition and metabolic markers were performed using GLMs. Tables 3 and 4 show the GLMs for the outcomes that presented significant effects. We found an effect of the Pro12Ala polymorphism on the percentage of body fat (%BF), fat-free mass (FFM) and lean mass (LM) (Table 3). Additional comparison between genotypes showed that Ala carriers in the OO and DieTBra+OO groups had a greater reduction in %BF than did ProPro homozygotes. FFM and LM showed significant increases for Ala carriers from the OO and DieTBra+OO groups compared to those for ProPro homozygotes. No differences were found between genotypes in the DieTBra group (Fig. 2).

**Table 3:** Generalized linear models of the effects of the *PPARG2* Pro12Ala polymorphism on the body composition of severely obese individuals after a 12-week nutritional intervention

Variables	Weight change			Percentage body fat change			Fat free mass change			Lean mass change		
	Adj. β*	95% CI	*P*	Adj. β*	95% CI	*P*	Adj. β*	95% CI	*P*	Adj. β*	95% CI	*P*
Intercept	3.504	−4.216, 11.224	0.374	0.432	−3.895, 9.115	0.432	−3.597	−11.462, 4.268	0.370	−3.593	−11.783, 4.597	0.390
Age	0.031	− 0.063, 0.125	0.517	− 0.056	− 0.125, 0.012	0.105	0.067	− 0.015, 0.193	0.110	0.071	−0.015, 0.157	0.107
Gender												
Females	0	Reference		0	Reference		0	Reference		0	Reference	
Males	−2.354	−4.741, 0.034	0.053	−0.010	− 2.450, 2.429	0.993	1.105	−1.844, 4.054	0.463	1121	− 1.950, 4.192	0.474
Baseline BMI	−0.060	−0.198, 0.078	0.397	0.006	−0.138, 0.150	0.938	0.019	−0.155, 0.193	0.827	0.015	−0.166, 0.197	0.868
Sedentary time change	0.007	−0.006, 0.020	0.270	0.002	−0.007, 0.011	0.610	0.001	−0.010, 0.012	0.876	0.001	−0.010, 0.012	0.856
Nutritional intervention												
OO	0	Reference		0	Reference		0	Reference		0	Reference	
DieTBra	−5.001	−7.135, −2.867	< 0.001	0.110	−1.430, 1.650	0.889	−1.390	−3.252, 0.472	0.143	−1.510	−3.448, 0.429	0.127
DieTBra+OO	−4.397	−6.474, −2.321	< 0.001	0.001	−1.468, 1.468	1.000	−1.502	−3.277, 0.273	0.097	−1.574	−3.423, 0.274	0.095
Pro12Ala polymorphism												
ProPro	0	Reference		0	Reference		0	Reference		0	Reference	
12Ala carriers	0.508	−1.823, 2.839	0.669	−2.338	−4.067, −0.608	0.008	2.917	0.825, 5.008	0.006	3.122	0.945, 5.301	0.005

Dependent variables: 12thweek measurement – baseline measurement. *OO* olive oil group, *DieTBra* traditional Brazilian diet group, *DieTBra + OO* traditional Brazilian diet plus olive oil group, *BMI* Body Mass Index

*General linear models were adjusted for age, gender, baseline BMI and change in sedentary time. When interactions were tested (SNP x nutritional intervention, gender x nutritional intervention), the *P* value was not significant (*P* > 0.05). Data for the outcome fat mass was not significant

**Table 4:** Generalized linear models of the effects of the *IL6* -174G/C polymorphism on the body composition of severely obese individuals after a 12-week nutritional intervention

Variables	Weight change			Fat mass change		
	Adj. β*	95% CI	*P*	Adj. β*	95% CI	*P*
Intercept	3.969	− 4.212, 11.348	0.369	−0.754	−9.145, 7.363	0.860
Age	0.026	−0.068, 0.121	0.585	−0.027	−0.112, 0.586	0.541
Gender						
Females	0	Reference		Reference		
Males	−2.411	−4.752, −0.070	0.044	1.281	−1.725, 4.286	0.404
Baseline BMI	−0.053	−0.188, 0.081	0.437	0.071	−0.110, 0.253	0.442
Sedentary time change	0.008	−0.005, 0.021	0.235	0.003	−0.008, 0.142	0.588
Nutritional intervention						
OO	0	Reference		Reference		
DieTBra	−4.980	−7.103, −2.858	< 0.001	−1.495	−3.390, 0.400	0.122
DieTBra+OO	−4.406	−6.478, −2.335	< 0.001	−1.975	−3.798, −0.153	0.034
-174G > C polymorphism						
GG	0	Reference		Reference		
C carriers	−0.219	−1.956, 1.519	0.805	−0.015	−1.513, 1.484	0.985

Dependent variables: 12^th^ week measurement – baseline measurement. *OO* olive oil group, *DieTBra* traditional Brazilian diet group, *DieTBra + OO* traditional Brazilian diet plus olive oil group, *BMI* Body Mass Index

*General linear models were adjusted for age, gender, baseline BMI and change in sedentary time. When interactions were tested (SNP x nutritional intervention, gender x nutritional intervention), the *P* value was not significant (*P* > 0.05). Data for the outcomes fat-free mass, body fat percentage and lean mass were not significant

DieTBra and DieTBra+OO promoted changes in weight and body composition that were independent of the polymorphisms. Considering the models for the Pro12Ala polymorphism, the weight reduction was greater for DieTBra and DieTBra+OO than for OO (Table 3). Considering the models for -174G > C polymorphism, the weight reduction was greater for DieTBra and DieTBra+OO than for OO, but the results were not independent of sex. Additionally, fat mass showed a greater reduction for DieTBra+OO than for OO (Table 4).

The nutritional interventions and the polymorphisms did not affect metabolic parameters. Data for the changes in all body composition and metabolic parameters according to the dietary intervention are shown in the supplemental material (Additional file 1: Table S1).

## Discussion

The key finding of the current study is the significant association between the Pro12Ala (rs1801282) polymorphism and body composition changes in severely obese patients after a 12-week intervention with extra virgin olive oil. Ala carriers that consumed extra virgin olive oil with or without DieTBra had a greater reduction in %BF and had a greater increase in FFM and LM than did ProPro homozygotes, while DieTBra had the same effect on ProPro homozygotes as it did on Ala carriers. To our knowledge, this is the first study demonstrating a synergistic effect of extra virgin olive oil intake and the Pro12Ala polymorphism on %BF, FFM and LM. These findings contribute to expand the knowledge about gene-diet interactions, showing that extra virgin olive oil may modulate favourable changes in body composition in the presence of the Ala allele of the *PPARG2* Pro12Ala polymorphism.

In our study, Ala carriers had greater reduction in %BF and a greater increase in FFM and LM than ProPro homozygotes when consuming extra virgin olive oil. The few studies that have assessed the effect of nutritional interventions on body composition parameters regarding the Pro12Ala polymorphism did not demonstrate a significant association. A study with obese individuals who received a low-energy diet and were followed up for 1 year found no association between this polymorphism and changes in FFM and fat mass [15], while other weight loss intervention studies with overweight subjects also found no significant impact of this polymorphism on %BF, FFM, and LM [36–39]. Individuals with a BMI between 25 and 40 kg/m^2^ under a behavioural treatment programme for obesity based on a MedDiet did not exhibit any effect of the polymorphism on %BF [40]. Our novel findings on the association of the Pro12Ala polymorphism with body composition parameters in severely obese individuals are important for the research field; however, larger sample sizes are necessary to confirm this result.

Anti-inflammatory mechanisms promoted by olive oil consumption might have had a role in the %BF-, FFM-, and LM-related results of the current study. Obesity is considered a low-grade chronic inflammatory state [41]. Adipose tissue inflammation seems to have a dominant role in the development of sarcopenia, eventually leading to skeletal muscle inflammation and dysfunction [42]. Nutrigenomic studies with short-term and sustained olive oil consumption have shown the upregulation of genes related to an anti-inflammatory profile and the downregulation of genes related to inflammation [43–45]. We hypothesize that the improved anti-inflammatory profile promoted by the olive oil intake may have affected the cross-talk between fat and muscle, thus favouring the signalling pathways of skeletal muscle synthesis [42]. Additionally, even when weight loss was not significant, such as in the OO group, olive oil was able to promote favourable changes in the body composition of Ala carriers.

Another aspect regarding skeletal muscle inflammation and dysfunction refers to weight cycling. Obese individuals trying to lose weight usually experience periods of weight loss followed by periods of weight regain, leading to inadequate changes in FFM and LM, thus favouring the development of sarcopenia [46]. Therefore, it seems that weight loss intervention with olive oil is promising for improving body composition in Ala carriers. However, further investigation is needed to clarify the precise mechanisms underpinning the influence of olive oil on LM and FFM in severely obese individuals.

Interactions between diet and body composition changes related to the Pro12Ala polymorphism, such as those observed in the current study, have not been reported to date. Associations have been found in observational studies between a higher P:S ratio and MUFA intake and a lower BMI and %BF [40, 47, 48]. Intervention studies have reported an interaction between a higher P:S ratio and a greater reduction in visceral adipose tissue [49], and between a higher total fat intake (≥ 42.6% of energy) and lower amount of weight loss [40]. Possibly, the interactions found in our study may be related not only to MUFA and PUFA but also to the antioxidant activity of phenolic compounds in olive oil, such as hydroxytyrosol and oleuropein; this possibility needs further investigation [45].

Our results have demonstrated that DieTBra and DieTBra+OO promoted a significant weight change that was independent of polymorphisms. Previous studies showed that a traditional Brazilian diet pattern was associated with a lower risk of overweight and obesity [18–20, 50]. Evidence has suggested that olive oil consumption even outside the Mediterranean dietary pattern may improve health parameters [51], and olive oil consumption also promotes the higher consumption of vegetables, thereby contributing to a healthier eating pattern [52]. Olive oil consumption has been increasing among Brazilians [21], and as olive oil is easily incorporated into the diet, such consumption may be encouraged as part of a healthy dietary pattern.

The current study has several strengths, such as the use of an advanced technique, namely, DXA, to determine body composition, the objective measurement of sedentary time and MVPA using a tri-axial accelerometer, the high retention rate, the real-world setting and intervention, and the randomized design. However, our study is not without limitations. These limitations include the sample size, especially the low number of individuals with the rare homozygote; the possibility of false-positive findings due to multiple comparisons; and the possibility of the under-reporting of food intake.

## Conclusions

In summary, our findings highlight that extra virgin olive oil intake may modulate favourable body composition changes, thus decreasing %BF and increasing LM and FFM in severely obese patients, even without weight loss, in the presence of the Ala allele of the Pro12Ala polymorphism. The beneficial effect of this gene-diet interaction may support the increasing body of evidence supporting the development of personalized interventions based on the genotype of the patient for more effective outcomes in the treatment of severe obesity.

## Additional file

Additional file 1: **Table S1.** Changes in the body composition and metabolic parameters after 12 weeks of nutritional intervention. (DOCX 66 kb)

## Abbreviations

%BF: Percentage of body fat
ANOVA: Analysis of variance
BMI: Body mass index
DieTBra: Traditional Brazilian diet
DieTBra+OO: Traditional Brazilian diet plus extra virgin olive oil supplementation
DRI: Dietary reference intake
DXA: Dual-energy X-ray absorptiometry
FFM: Fat-free mass
GLM: General linear model
HbA1c: Haemoglobin A1c
HDL: High-density lipoprotein
HOMA-IR: Homeostasis model assessment of insulin resistance
LDL: Low-density lipoprotein
lL-6: Interleukin-6
LM: Lean mass
MUFA: Monounsaturated fatty acids
MVPA: Moderate-to-vigorous physical activity
OO: Olive oil supplementation
P:S ratio: Polyunsaturated:saturated fatty acids ratio
PUFA: Polyunsaturated fatty acids
qPCR: quantitative polymerase chain reaction
SUS: Brazilian Unified Health System
