# Supplementary material for: PPARG2 Pro12Ala polymorphism influences body composition changes in severely obese patients consuming extra virgin olive oil: a randomized clinical trial
Source: Nutr Metab (Lond). 2018 Jul 17;15:52. doi: 10.1186/s12986-018-0289-4 (PMC6050653; doi:10.1186/s12986-018-0289-4)
Supplement: Supplementary file 1 — Table S1. Changes in the body composition and metabolic parameters after 12 weeks of nutritional intervention. (DOCX 66 kb) [file 12986_2018_289_MOESM1_ESM.docx]

**Additional file 1: Table S1.** Changes in the body composition and metabolic parameters after 12 weeks of nutritional intervention

| Variable | OO  N=43 | DieTBra  N=43 | DieTBra+OO  N=47 | P value* |
| --- | --- | --- | --- | --- |
| Δ Weight (kg) | 0.95 ± 3.60 | -3.10 ± 5.27 | -2.53 ± 3.62 | <0.001 |
| Δ Fat mass (kg)^†^ | 1.16 ± 2.72 | -0.34 ± 3.47 | -0.45 ± 2.90 | 0.039 |
| Δ Fat free mass (kg)^†^ | -0.29 ± 3.03 | -0.74 ± 3.27 | -0.67 ± 2.90 | 0.707 |
| Δ Body fat percentage (%)^†^ | 0.64 ± 2.43 | 0.28 ± 2.60 | 0.13 ± 2.25 | 0.579 |
| Δ Lean mass (kg) ^†^ | -0.33 ± 3.16 | -0.86 ± 3.38 | -0.74 ± 3.04 | 0.684 |
| Δ Fasting glucose (mg/dL) | -1.29 ± 18.27 | -5.54 ± 23.48 | -3.06 ± 31.29 | 0.668 |
| Δ Fasting insulin (μU/mL) | -0.23 ± 16.70 | 0.67 ± 13.53 | 0.99 ± 10.08 | 0.809 |
| Δ HOMA-IR | -0.31 ± 5.39 | -0.20 ± 3.83 | 0.54 ± 4.63 | 0.709 |
| Δ HbA1c (%) | 0.09 ± 0.87 | -0.32 ± 1.52 | -0.03 ± 1.70 | 0.212 |
| Δ Total cholesterol (mg/dL) | -5.51 ± 30.45 | -6.56 ± 28.39 | -1.40 ± 36.66 | 0.656 |
| Δ HDL cholesterol (mg/dL) | 1.91 ± 8.17 | 0.46 ± 8.90 | 1.60 ± 8.35 | 0.361 |
| Δ LDL cholesterol, (mg/dL) | -5.12 ± 21.79 | -4.27 ± 23.84 | -1.94 ± 33.61 | 0.921 |
| Δ Triglyceride (mg/dL) | -6.67 ± 41.36 | -14.56 ± 51.20 | -6.55 ± 41.59 | 0.717 |

Data are presented as unadjusted means ± SD. OO: olive oil, DieTBra: traditional Brazilian diet, DieTBra+OO: traditional Brazilian diet plus olive oil, HOMA-IR: homeostatic model assessment for insulin resistance, HbA1c: haemoglobin A1c, HDL: high density lipoprotein, LDL: low density lipoprotein.

*Kruskall-Wallis test

^†^OO: n=28, BraDiet: n=32, BraDiet+OO: n=37
